# Leveraging Geospatial Approaches to Characterize the HIV Prevention and Treatment Needs of Out-of-School Adolescent Girls and Young Women in Ethiopia

**DOI:** 10.1007/s10461-019-02537-1

**Published:** 2019-05-27

**Authors:** Y. Wang, C. A. Comins, A. Mulu, S. A. Abebe, K. Belete, T. T. Balcha, S. Baral, S. R. Schwartz

**Affiliations:** 1 Department of Epidemiology, Johns Hopkins Bloomberg School of Public Health, 615 N Wolfe St E7003, Baltimore, MD 21231 USA; 2 Armauer Hansen Research Institute (AHRI), Addis Ababa, Ethiopia; 3 United States Agency for International Development, Addis Ababa, Ethiopia

**Keywords:** Adolescent girls and young women, Epidemiology, Out of school, Ethiopia, Time location sampling, Venue-based sampling, HIV

## Abstract

Adolescent girls and young women (AGYW) remain underserved and at risk for HIV acquisition in Ethiopia. However, there is significant risk heterogeneity among AGYW with limited consensus on optimal strategies of identifying vulnerable AGYW. This study assessed the utility of venue-based sampling approaches to identify AGYW at increased risk for HIV infection. Venue mapping and time-location-sampling (TLS) methods were used to recruit AGYW from three sub-cities of Addis Ababa, February–June 2018. Interviewer-administered surveys captured socio-demographic and behavioral characteristics. Measures of AGYW vulnerability were assessed geographically and described by venue type. A total of 2468 unique venues were identified, of which 802 (32%) were systematically selected for validation and 371 (46%) were eligible including many sites that would traditionally not be included as venues in need of HIV prevention services. Overall, 800 AGYW were enrolled across 81 sampled venues. AGYW reached were largely out-of-school (n = 599, 75%) with high proportions of AGYW reporting transactional sex (n = 101, 12.6%), food insecurity (n = 165, 20.7%) and migration (n = 565, 70.6%). Taken together, these data suggest the utility of TLS methods in reaching vulnerable, out-of-school AGYW in Addis Ababa, Ethiopia.

**Electronic supplementary material:**

The online version of this article (10.1007/s10461-019-02537-1) contains supplementary material, which is available to authorized users.

## Introduction

Adolescent girls and young women (AGYW) are well understood to be at high risk of incident HIV infections in Southern and Eastern Africa [1]. Data more broadly from across Sub-Saharan Africa have consistently confirmed the importance of effectively addressing the needs of AGYW as part of a comprehensive HIV response [2–6]. Intersecting biological, economic, and structural determinants have been shown to potentiate HIV risks among AGYW [3–5]. However, AGYW constitute a large and varied population and there have been limited studies of strategies to effectively reach AGYW at heightened vulnerability for HIV.

The HIV epidemic in Ethiopia varies from that of other East African countries with an overall HIV prevalence among adults aged 15–49 of 0.9% [7]. However, HIV prevalence varies significantly by age, sex, and location, with cisgender women being disproportionately affected [7]. In Addis Ababa, the HIV adult prevalence is estimated to be 3.4% [7], while HIV surveillance data from antenatal clinics estimate that 2.3% of sexually active AGYW are living with HIV [2]. Available data suggest that women in Ethiopia tend to have earlier sexual debut compared to men, with less reported use of condoms [7]. Furthermore, young women migrating to urban areas in Ethiopia have reported challenging working and living conditions including coerced sex and high probabilities of entering formal sex work at young ages, reinforcing HIV risks [7–11]. Better serving the HIV prevention, and increasing HIV treatment needs of AGYW in Ethiopia necessitates more granular data of the specific and modifiable HIV-related vulnerabilities.

The Determined, Resilient, Empowered, AIDS-free, Mentored and Safe (DREAMS) program being implemented across Sub-Saharan Africa (SSA) has focused on effectively scaling the HIV prevention needs of AGYW across SSA. To date, there has been no reported consensus of sampling approaches for AGYW. Previous studies and programs have often relied on school-based recruitment strategies or household surveys to identify AGYW [12]. However, school and household-based approaches may undersample the most vulnerable AGYW, including those not enrolled or in frequent attendance at school, or who do not have stable housing. Furthermore, while evaluations of sampling methods for reaching AGYW are limited, the effect of sampling methods on recruitment of other key populations, such as female sex workers or men who have sex with men, are increasingly being assessed [3, 4].

There is no universal method for identifying and recruiting populations vulnerable to HIV [13, 14]. Respondent driven sampling (RDS), an incentive-based peer-referral sampling method, has been demonstrated to effectively reach hidden and vulnerable populations [15–18]. Another method for accessing hard-to-reach populations is time-location sampling (TLS), a form of venue-based sampling [19]. TLS consists of recruiting participants from specific geographic locations, or venues, at predetermined times when the population of interest are likely to be present [20]. TLS has proven successful at recruiting key populations across a number of countries and settings [21–26]. Varying sampling approaches have demonstrated significant differences in the risk strata of those recruited and in turn those potentially reached for prevention interventions [27–29].

Reaching vulnerable AGYW is critical for effective HIV prevention programming, including AGYW who may be along a pathway of risk in which vulnerabilities can be addressed and HIV-related risk trajectories altered [12]. Household sampling frames may not adequately capture AGYW with increased vulnerability, while traditional sex work hotspot recruitment alone will reach only AGYW at highest risk [30]. Comprehensive venue-based mapping and TLS may offer a promising approach for reaching a geographically and situationally diverse sample of vulnerable AGYW [31]. Thus, the objective of this paper is to assess the potential of a systematic and community-informed venue-based sampling approach to identify and reach a diverse sample of vulnerable AGYW.

## Methodology

### Study Design, Setting and Population

This was a venue-based sampling study of AGYW in Addis Ababa, Ethiopia. The study was implemented in three sub-cities of Addis Ababa purposively selected based on geographic diversity and venue type distribution: Addis Ketema, Kolfe Keranio, and Akaki Kality. Addis Ketema, with a population of 271,644 and an area of 7.4 km^2^ [32], is a densely populated sub-city in Addis Ababa with many bars and hotels. Akaky Kaliti has a population of 195,273 and spans an area of 118 km^2^ [33]. It is the industrial and manufacturing area of Addis Ababa, with a high concentration of construction sites and factories. Kolfe Keranio, with an area of 61.3 km^2^ and population size of 546,219 [34], is located on the western side of Addis Ababa and stretches from the southern to northern part of the city, encompassing transportation hubs along the corridor, along with factories, restaurants and bars. Sub-cities were chosen to maximize geographic and business diversity. Eligible AGYW were 15–24 years old, attending a selected venue and speaking Amharic or English.

### Venue Mapping, Sampling and Recruitment

Venue mapping combined with time location sampling (TLS) were utilized to identify and sample venues frequented by AGYW [26]. With support from local stakeholders, local HIV prevention control officers, and an AGYW community advisory group (CAG), AGYW venues were identified through in-person discussions with community-based key informants (KIs) and compiled into an electronic database. KIs included a variety of community leaders and other individuals in contact or knowledgeable about where out-of-school AGYW work and/or frequent (e.g. urban health workers, church leaders, taxi drivers, street vendors, bar or restaurant owners, etc.) and in- and out-of-school AGYW. Discussions were informal and occurred at purposively selected locations throughout the sub-cities. A subset of venues was selected for on-site verification and enumeration, and a sampling calendar for recruitment was developed. Venue sampling followed standard TLS methods, based on the Priorities for Local AIDS Control Efforts (PLACE) [35]. Within this study, a venue was defined as a geographic location or physical space where AGYW work and/or congregate.

#### Venue Identification

To identify venues frequently attended by vulnerable, out-of-school AGYW, community-based KIs were consulted until no new venues were identified. KIs were identified based on their knowledge of and/or experience working with AGYW and selected based on a matrixed set of predetermined characteristics, including representation from the governmental sector, economic sector (e.g., market sellers and business owners), and non-governmental organizations (e.g., those who work in areas of HIV and sexual health, adolescent health, migration, orphans, and/or vulnerable children). The purpose of the KI discussions was to introduce the study and identify up to 10 venues where predominantly out-of-school AGYW attend. When no new venues were identified, the team began deduplication of the venue database. This process collated unique venues into a list and accounted for how many times a particular venue was mentioned in the identification process.

#### Venue Verification and Enumeration

A subset of venues from each sub-city was selected for validation and enumeration. The number of venues selected for verification was based on the total number identified per sub-city. If 200 or fewer venues were identified, all venues (100%) were validated; if more than 200 were identified, a power function was utilized such that as the number of venues per sub-city increased, the total venues validated also slowly increased (y = 61.684x^0.778^). Venues mentioned five or more times by KI were considered high priority and automatically included for validation. Of the remaining venues, venues were then randomly sampled proportional to the venue type (e.g. bars/restaurants, broker houses, brothels, etc.). For example, if 10% of venues identified by KIs were bars or restaurants, we stratified sampling to ensure that approximately 10% of the total venues randomly selected for validation were bars or restaurants. The exception to this rule was smaller venue types (comprising less than 3% of venues); to ensure representation of smaller venue types we selected all venues from these types for validation.

For selected venues, validation and enumeration were conducted on-site by a local data collection team to validate the venues’ existence, location, venue type, hours of operation, number of AGYW, peak attendance of AGYW, safety, gatekeepers, and contact information. Validation was performed with a person(s) knowledgeable about the venue (e.g., owner, manager, other employees or gatekeepers). GPS waypoint coordinates captured venue location on Garmin GIS devices (WGS 84 coordination system) [36]. Data collectors counted the total number of AGYW physically present at the venue over a 30-min period. Typically, two field workers were assigned to visit one venue, with one sitting by the location of entry, counting AGYW in the main area or AGYW entering the venue during the 30-min period. The other field worker walked around the entirety of the venue to ensure all AGYW were counted and to gain additional information from the venue manager or gatekeeper.

Venues with more than eight AGYW per hour on-site were eligible for sampling [35]. At least eight AGYW per hour ensured AGYW frequented the venue and preserved the minimum effective yield during a given sampling event to maximize productivity. Eligibility criteria for venues to be included in the final venue sampling frame were: (1) majority of AGYW attending venue were out-of-school; (2) four or more AGYW observed during a 30-min observation period at the venue; (3) venue deemed safe by data collection team; and (4) key gatekeepers were accepting of study activities. The AGYW CAG facilitated validation, helping to locate venues, grant entry, and build rapport. To assess whether the majority of AGYW were out-of-school, the study team observed whether AGYW were wearing school uniforms (if verification activities occurred during school hours) and discussed with AGYW and venue managers present at the venue during data collection.

#### Venue Selection

All eligible, enumerated venues with at least one 4-h venue-specific-day-time (VDT) block were included in the final sampling frame. VDT blocks were generated from the hours when AGYW were found at the venue and per discussions with the venue manager and AGYW during the verification phase. VDT blocks were randomly selected without replacement and populated into monthly sampling calendars. For example, if venue A is frequented by AGYW from 14:00 to 22:00 on Monday through Friday, this would result in 10 VDT blocks for inclusion in the sampling frame: 14:00–18:00 and 18:00–22:00 each day on Monday through Friday.

#### Recruitment

Recruitment occurred from February to June 2018. All AGYW found at the selected VDT block during the 4-h period were systematically approached and informed of the study, screened for eligibility, and offered participation if eligible. A maximum of 15 AGYW per site were recruited to ensure that one venue did not overpopulate the sample. The total number of AGYW enrolled per site may have been less than the total interested and eligible based on time constraints of the 4-h block.

### Sample Size Calculations

Sample size was determined based on HIV prevalence estimates from antenatal care-based sentinel HIV surveillance, Demographic and Health Survey data (2016), and Ethiopian education enrollment data [37–39]. Overall, a 5% HIV prevalence among out-of-school AGYW was hypothesized, which is approximately twice as high as that among all sexually active age-matched AGYW based on antenatal surveillance data; an estimated 80% of AGYW sampled were expected to be out-of-school based on local estimates among AGYW as well as purposive sampling methods to only include venues with at least 50% of the AGYW attending being out-of-school [38]. Thus, to estimate HIV prevalence with a hypothesized prevalence of 5% and a precision of ± 1.5%, a sample size of 800 AGYW in Addis Ababa was determined.

The sample size for each sub-city in Addis Ababa was derived based on the total number of venues identified by KIs, the proportion of enumerated venues eligible, and the estimated total eligible venues. The sub-city targeted sample size of the selected three sub-cities of Addis was 187 for Kolfe Keranio, 336 for Addis Ketema, and 277 for Akaki Kality.

### Study Procedures and Measures

Eligible, consenting AGYW were enrolled and administered a socio-behavioral questionnaire in a private space. Questionnaires covered demographic characteristics, vulnerability measures, health seeking behaviors, and prevention indicators. Demographic characteristics assessed age, ethnicity, education, literacy, living situation, and marital status, among others. Vulnerability measures included migration history, adult support, food security, alcohol consumption, age of sexual debut, history of physical and sexual abuse, history of STI symptoms, and engagement in transactional sex [40–48]. Food security was classified into no food insecurity (i.e., did not go to sleep hungry in past 4 weeks), yes-rare (i.e., went to sleep hungry once or twice a week in past 4 weeks), and yes-sometimes or often (i.e., went to sleep hungry three times or more in past 4 weeks) [49]. HIV prevention indicators included condom use, prior testing for HIV, and prior STI treatment. Condom usage, prior testing for HIV, and history of STI symptoms and STI treatment were asked within the preceding 12 months.

### Analyses

Data were collected and managed on tablets using RedCap™ [50] and analyzed using Stata version 15.0 (College Station, Texas) [51]. Descriptive analyses examined AGYW demographics, vulnerability, and preventative behaviors across venue types. AGYW venues were categorized into eight venue types based on input from the Ethiopian investigative and data collection teams: (1) bar, restaurant; (2) hotel, hostel, guest house; (3) brokers’ place [i.e., places connecting AGYW to different jobs such as domestic worker or waitress]; (4) street, street corner, street market, main transportation center; (5) construction site, shed, factory; (6) special villages [i.e., a hybrid of living places and brothels]; (7) youth and training centers; and (8) other [i.e., cinemas/video shops, kolo (barley) preparation sites, and dumping sites].

Although the objective of this paper was to describe vulnerability of AGYW recruited through a venue-based approach, we also compared the prevalence of vulnerability across venue types as a sensitivity analysis to assess the effectiveness of the TLS approach (e.g. if resources were commonly being allocated to venues in which minimal vulnerability was found, the approach may not be efficient at reaching vulnerable AGYW). Thus, logistic regression models clustering on venues to account for non-independence within venues, were run to assess associations of the venue types with five key outcomes that are markers of vulnerability or engagement in HIV prevention, including being out-of-school, having migrated into Addis Ababa, HIV testing history (tested in past 12 months), food insecurity (ever vs. never in past 4 weeks), and engagement in transactional sex.

Finally, ArcGIS version 10.5 [52] mapped AGYW verified venues using GPS coordinates and heat maps illustrated the prevalence of factors amplifying vulnerability or prevention of AGYW recruited from specific venues at the Woreda level, the subdivision of sub-cities.

### Ethical Considerations

The study was approved by the Johns Hopkins School of Public Health Institutional Review Board in Baltimore, Maryland, USA, the AHRI/ALERT Ethics Review Committee in Addis Ababa, Ethiopia, and the Ethiopian National Research Ethics Review Committee. All participants provided written informed consent; AGYW ages 15–17 years were considered as emancipated minors as per the Ethiopian National HIV and Testing Guideline [53]. Staff were trained in protection of human subjects in research, study protocol, and standard operating procedures.

## Results

**Fig. 1:**
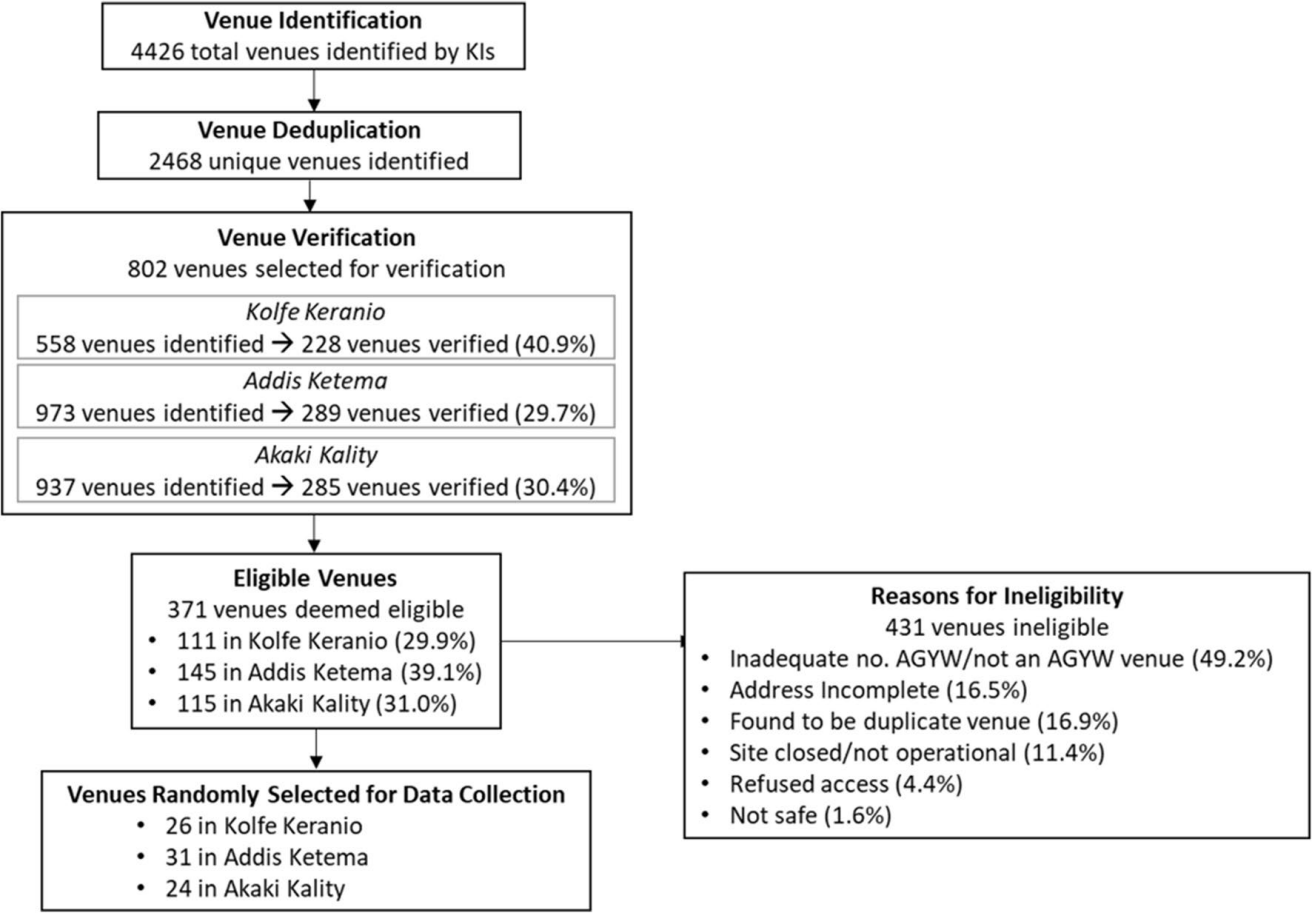
Flow chart of venue mapping

**Fig. 2:**
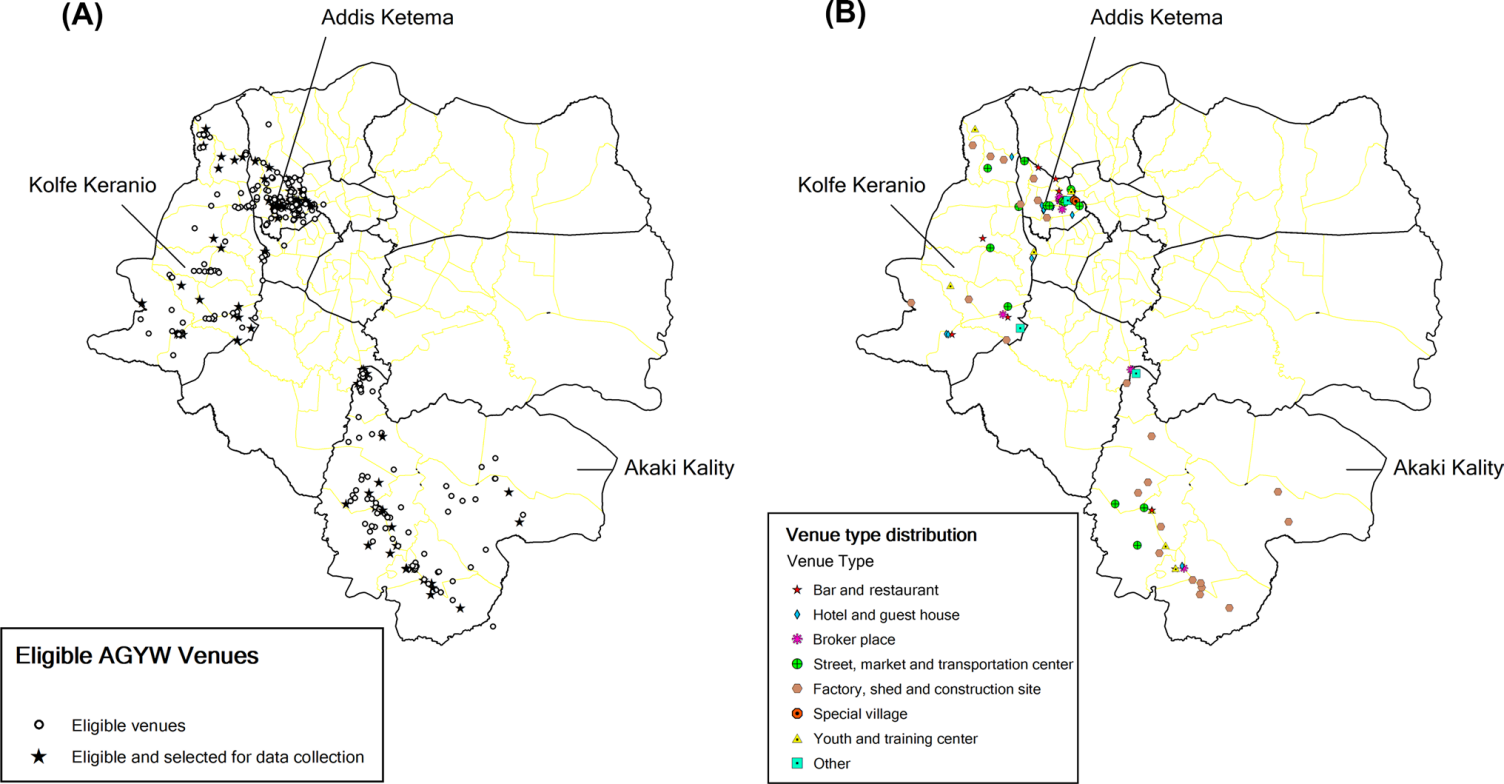
Mapping of validated and selected venues in three sub-cities in Addis Ababa, Ethiopia. Maps illustrate **a** eligile and selected venues across and **b** distribution of selected venues by venue type

Of 2468 unique venues identified through 688 KI discussions, 802 venues were selected for verification and, of these, 371 were determined to be eligible (Fig. 1). Of the 371 eligible venues, 81 were randomly sampled for recruitment (26 from Kolfe Keranio, 31 from Addis Ketema and 24 Akaki Kality). Figure 2 maps the eligible and sampled venues by geolocation (Fig. 2a), as well as sampled venues by venue type (Fig. 2b).

A total of 1882 AGYW were screened of which 800 (42%) AGYW were eligible and enrolled from the 81 randomly sampled VDT blocks across the three sub-cities; 467 (25%) were ineligible and 615 (33%) refused. Among enrolled AGYW, nearly a third (32%, n = 254/800) of AGYW were recruited from construction sites or factories; 19% (n = 148/800) from streets, markets, and transportation centers; 14% (n = 112/800) from hotels and guest houses; 10% (n = 81/800) from brokers’ places; 9% (n = 75/800) from bars/restaurants; 8% (n = 67/800) from youth and training centers; 4% (n = 33/800) from special villages; and 4% (n = 30/800) from other venue types. Bars/restaurants and hotels had amongst the highest proportion of enrollments among AGYW screened at 69% and 66%, respectively (Supplemental Table I). The “other” venue type and special villages were found hardest to recruit from, with 16% and 35% of screened AGYW enrolled, respectively. Reasons for low screening to enrollment numbers included ineligibility due to language barriers, unwillingness to participate in an HIV-related study, lack of time, and inability to enroll all eligible AGYW due to insufficient private interview space during the 4-h block and other logistical considerations.

Of the 800 AGYW participants, 182 were enrolled in Kolfe, 337 in Addis Ketema and 281 in Akaki Kality. The mean age of AGYW was 20.1 years (sd: 2.5) and 75% (n = 596/791) were currently out-of-school. Overall, 11% (n = 87/798) of AGYW were married and 63% (n = 502/799) reported ever having sex. Demographic characteristics by venue type of recruitment are presented (Table 1).

**Table 1:** Demographics of adolescent girls and young women by venue type, n = 800, Addis Ababa, Ethiopia

	Bar, restaurant (n = 75)		Hotel, hostel, guest house (n = 112)		Broker house (n = 81)		Street, market, transportation (n = 148)		Factory, construction (n = 254)		Special village (n = 33)		Youth center (n = 67)		Other (n = 30)		Total (n = 800)	
	n	%	n	%	n	%	n	%	n	%	n	%	n	%	n	%	n	%
Sub-city																		
Addis Ketema	35	46.7	69	61.6	50	61.7	88	59.5	34	13.3	33	100.0	13	19.4	15	50.0	337	42.1
Akaki Kaliti	21	28	24	21.4	22	27.2	29	19.6	151	59.0	0	0.0	24	35.8	11	36.7	281	35.1
Kolfe Keranio	19	25.3	19	17.0	9	11.1	31	20.9	71	27.7	0	0.0	30	44.8	4	13.3	182	22.8
Age																		
15–19 years old	27	36.0	40	35.7	43	53.1	87	58.8	90	35.4	9	27.3	27	40.3	11	36.7	344	41.8
20–24 years old	48	64.0	72	64.3	38	46.9	61	41.2	164	64.6	24	72.7	40	59.7	19	63.3	466	58.2
Ethnic group																		
Amhara	30	40.0	46	41.1	37	45.7	58	39.2	81	31.6	15	45.5	28	41.8	8	26.7	303	37.9
Guragie	7	9.3	30	26.8	8	9.9	27	18.2	49	19.3	4	12.1	7	10.4	7	23.3	139	17.4
Oromo	29	38.7	23	20.5	21	25.9	30	20.3	73	28.7	9	27.3	21	31.3	7	23.3	213	26.6
Tigray	5	6.7	6	5.4	3	3.7	10	6.8	11	4.3	1	3.0	5	7.5	2	6.7	43	5.4
Welaita	1	1.3	4	3.6	7	8.6	14	9.5	17	6.7	0	0.0	3	4.5	5	16.7	51	6.4
Other	3	4.0	3	2.7	5	6.2	9	6.1	23	9.1	4	12.1	3	4.5	1	3.3	51	6.4
Ever attended school	72	96.0	107	95.5	75	92.6	141	95.3	245	96.5	28	84.8	60	89.6	27	90.0	755	94.4
Education level																		
Primary 1 cycle (grades 1–4)	9	12.5	15	14.0	9	12.0	14	10.0	37	15.1	6	21.4	5	8.3	5	18.5	100	13.3
Primary 2 cycle (grades 5-8)	31	43.1	44	41.1	35	46.7	54	38.6	84	34.3	14	50.0	20	33.3	20	74.1	302	40.1
Secondary (9–10)/Preparatory (11–12)/Technical	19	26.4	30	28.0	23	30.7	48	34.3	78	31.8	5	17.9	29	48.3	2	7.4	234	31.0
College/University	13	18.1	18	16.8	8	10.7	24	17.1	46	18.8	3	10.7	6	10.0	0	0.0	118	15.6
Able to read	69	92.0	96	85.7	70	86.4	133	89.9	225	88.9	23	69.7	58	86.6	26	86.7	700	87.6
Own a mobile phone	67	89.3	102	91.1	70	86.4	114	77.6	235	92.5	27	84.4	50	74.6	26	86.7	691	86.6
Married	4	5.3	6	5.4	5	6.2	10	6.8	48	18.9	3	8.8	10	14.9	2	6.7	87	10.9
Housing																		
Renting place	28	36.8	49	43.8	25	30.9	74	50.0	161	63.4	12	36.4	24	35.8	20	66.7	393	49.1
Family home/own place	9	11.8	17	15.2	14	17.3	39	26.4	55	21.7	6	18.2	28	41.8	3	10.0	171	21.4
Staying at someone else’s place	17	22.4	38	33.9	38	46.9	21	14.2	28	11.0	10	30.3	5	7.5	6	20.0	163	20.4
Other	21	28.0	8	7.1	4	4.9	14	9.5	10	3.9	5	15.2	10	14.9	1	3.3	73	9.1
Living parents																		
Both parents living	47	62.7	79	70.5	56	69.1	106	71.6	192	75.6	18	54.5	46	68.7	21	70.0	565	70.6
One parent living	24	32.0	29	25.9	21	25.9	27	18.2	53	20.9	12	36.4	16	23.9	4	13.3	186	23.3
No parents living	4	5.3	4	3.6	4	4.9	15	10.1	9	3.5	3	9.1	5	7.5	5	16.7	49	6.1
Ever pregnant	13	17.3	23	20.5	17	21.0	27	18.2	48	19.0	8	24.2	15	22.4	13	43.3	164	20.5

Measures of vulnerability and engagement in HIV prevention behaviors were also described across venue types (Table 2). Overall, 71% (n = 566/800) of AGYW migrated into Addis Ababa from rural or peri-urban areas. Approximately 13% of AGYW lived in Addis Ababa for less than one year. Among the 63% (n = 502/799) of sexually active AGYW, 70% (n = 353/502) had their sexual debut under the age of 18. Half of the sexually active participants (n = 194/390) reported never using a condom and a further 31% (n = 120/390) reported inconsistent condom use. History of engagement in transactional sex was 13% (n = 101/800) and ranged from 5% among AGYW at factory and construction sites to 50% at “other” venue types, consisting largely of dumping grounds. A total of 69% (n = 553/799) of AGYW reported a prior HIV test, with a range of 61% among those at streets, markets and transportation centers, to 94% among AGYW recruited at special villages. A fifth, 21% (n = 165/799), of participants reported experiencing food insecurity in the past 4 weeks. In sensitivity analyses comparing vulnerability measures across venue types, no major differences in vulnerabilities were observed across venue types (results not shown).

**Table 2:** Vulnerability among adolescent girls and young women by venue type, n = 800, Addis Ababa, Ethiopia

	Bar, restaurant (n = 75)		Hotel, hostel, guest house (n = 112)		Broker house (n = 81)		Street, market, transportation (n = 148)		Factory, construction (n = 254)		Special village (n = 33)		Youth center (n = 67)		Other (n = 30)		Total (n = 800)	
	n	%	n	%	n	%	n	%	n	%	n	%	n	%	n	%	n	%
Migrated into Addis	55	73.3	84	75.0	65	80.2	84	56.8	195	76.8	21	63.6	42	62.7	20	63.3	565	70.6
Migrated from																		
City	10	17.9	17	20.2	13	20.0	23	27.4	25	12.8	3	14.3	4	9.5	5	26.3	100	17.7
Town	9	16.1	21	25.0	22	33.8	25	29.8	50	25.5	9	42.9	22	52.4	3	15.8	161	28.4
Rural area	36	65.5	46	54.8	30	46.2	36	42.9	121	61.7	9	42.9	16	38.1	11	57.9	305	53.9
Adult support																		
Emotionally	12	16.0	17	15.2	10	12.3	26	17.6	44	17.4	10	30.3	11	16.4	7	23.3	137	17.2
Financially	3	4.0	2	1.8	3	3.7	4	2.7	3	1.2	1	3.0	6	9.0	0	0.0	22	2.7
Both	34	45.3	58	51.8	32	39.5	77	52.0	141	55.7	10	30.3	39	58.2	10	33.3	401	50.2
Neither	26	34.7	35	31.3	36	44.4	41	27.7	65	25.7	12	36.4	11	16.4	13	43.3	239	29.9
Food insecurity																		
None	62	82.7	98	87.5	56	69.1	115	77.7	209	82.6	23	69.7	53	79.1	18	60.0	634	79.3
Yes, rarely	9	12.0	6	5.4	10	12.3	17	11.5	26	10.3	6	18.2	9	13.4	7	23.3	90	11.3
Yes, sometimes or often	4	5.3	8	7.1	15	18.5	16	10.8	18	7.1	4	12.1	5	7.5	5	16.7	75	9.4
Ever had sex	50	66.7	77	68.8	54	66.7	80	54.1	152	60.1	25	75.8	39	58.2	25	83.3	502	62.8
Age of Sexual debut																		
< 15 years old	8	16.0	5	6.5	7	13.0	19	23.8	11	7.2	6	24.0	5	12.8	5	20.0	66	13.1
15–18 years old	31	62.0	50	64.9	39	72.2	42	52.5	76	50.0	11	44.0	20	51.3	18	72.0	287	57.2
> 18 years old	11	22.0	22	28.6	8	14.8	19	23.8	65	42.8	8	32.0	14	35.9	2	8.0	149	29.7
Condom use (vaginal)																		
Never	10	27.8	25	39.7	23	57.5	37	52.9	77	68.8	7	35.0	12	40.0	3	15.8	194	49.7
Inconsistent use	15	41.7	25	39.7	8	20.0	20	28.6	24	21.4	10	50.0	13	43.3	5	26.3	120	30.8
Always	11	30.6	13	20.6	9	22.5	13	18.6	11	9.8	3	15.0	5	16.7	11	57.9	76	19.5
Transactional sex (money or goods)	18	24.0	15	13.4	6	7.4	17	11.5	12	4.7	10	30.3	8	11.9	15	50.0	101	12.6
Ever tested for HIV	56	74.7	82	73.2	52	64.2	90	60.8	168	66.4	31	93.9	50	74.6	24	80.0	553	69.2
Symptoms of STI in prior year	11	14.5	10	8.9	2	2.5	6	4.1	15	5.9	6	18.2	6	9.0	6	20.0	62	7.8
Ever experienced physical abuse	24	32.0	36	32.1	24	29.6	59	39.9	62	24.6	15	45.5	17	25.4	10	33.3	247	31.0
Ever experienced sexual abuse	9	12.0	13	11.6	16	19.8	21	14.2	34	13.4	5	15.2	8	11.9	4	13.3	110	13.8
Alcohol consumption																		
Never	33	44.0	54	48.2	51	63.0	90	60.8	168	66.4	17	51.5	31	46.3	12	40.0	456	57.1
Once a week or less	25	33.3	32	28.6	25	30.9	42	28.4	77	30.0	10	30.3	26	38.8	13	43.3	249	31.2
Twice or more per week	17	22.7	26	23.2	5	6.2	16	10.8	9	3.6	6	18.2	10	14.9	5	16.7	94	11.7

**Fig. 3:**
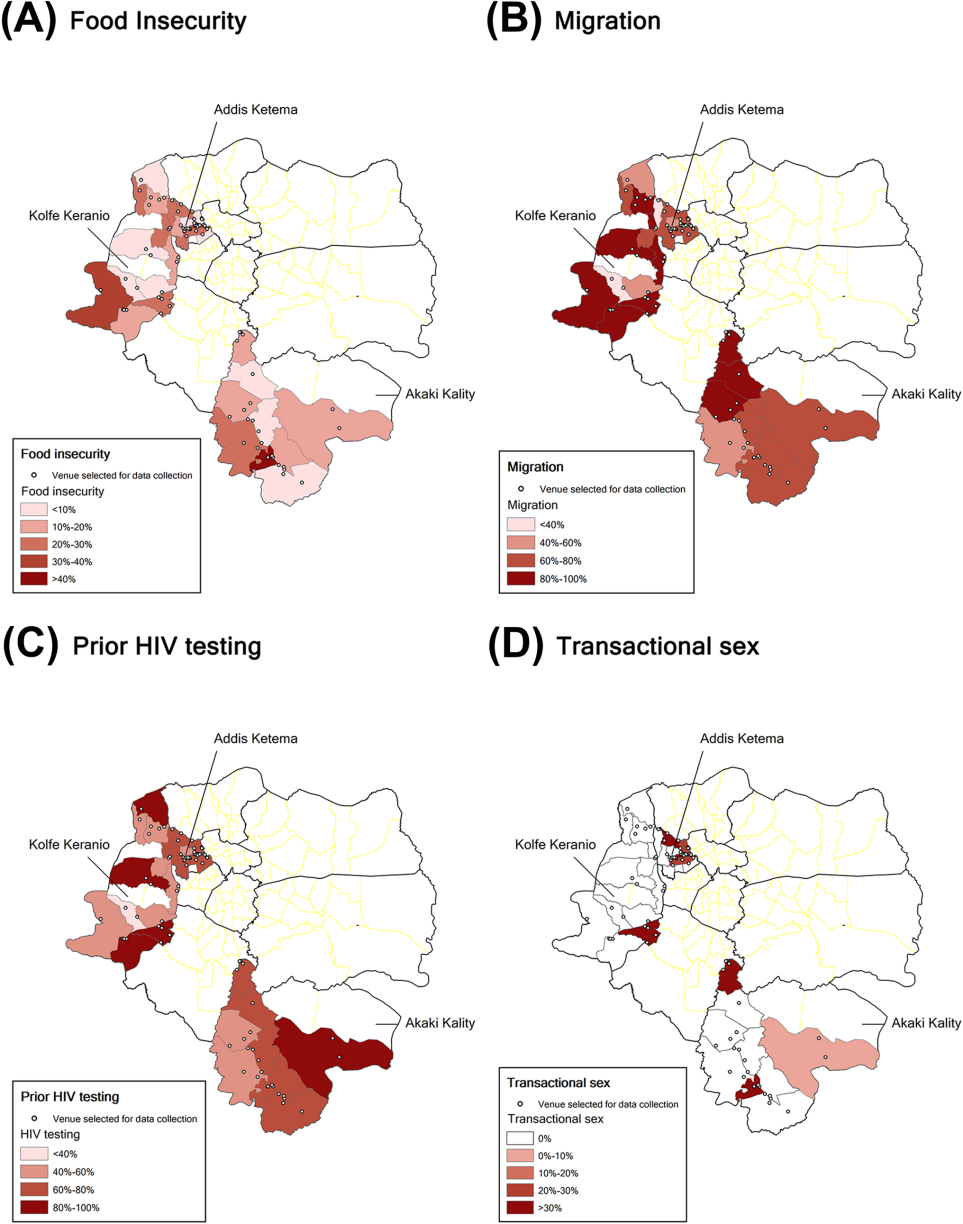
Spatial distribution of prevalence of HIV risk factors and prevention behaviors in across woredas in three sub-cities in Addis Ababa, Ethiopia. Maps illustrate the prevalence and distribution of **a** reported food insecurity in the past month; **b** history of migration from outside of Addis Ababa; **c** prior uptake of HIV testing; and **d** history of engagement in sex for goods or money

Heat maps were used to visualize vulnerabilities, including migration history, food insecurity and engagement in transactional sex, as well as HIV testing history among AGYW (Fig. 3). The geographic concentration of transactional sex among AGYW was found in the southeast part of Kolfe Keranio, southern and eastern pockets in Akaki Kality, and northeast part of Addis Ketema. Although the burden of vulnerabilities overlapped in some areas, risks were not entirely overlapping.

## Discussion

Venue-based TLS was an effective method to systematically identify and recruit predominantly out-of-school AGYW in Addis Ababa, Ethiopia. Vulnerability and risks for HIV infection were high across a broad array of venue types, reinforcing the value in recruiting at venues identified through a thorough community-informed mapping and sampling process. These data suggest that approaches focused on traditionally identified hotspots (such as brothels, guest houses, hotels, bars, and restaurants) may fail to reach and represent vulnerable AGYW across a diverse array of higher risk venues. Vulnerabilities, including engagement in transactional sex among AGYW, were reported across the spectrum of venue types. Furthermore, geospatial mapping can be used to refine and prioritize areas for future HIV prevention programming in order to optimize resource allocation.

TLS led to the identification of a substantial number of venues, resulting in a diverse sample of geographic locations, venue types, and AGYW in Addis Ababa. Out-of-school AGYW have been observed to be predominantly migrants and at heighted risk for HIV [8], and three quarters of AGYW reached through this venue-based approach were out-of-school. Although venue-based approaches have been used commonly to enroll female sex workers and other key populations at risk for HIV, their application to reach diverse samples of adolescent girls is more recent. A study employing the PLACE Method in Zimbabwe successfully utilized venue-based sampling methods to reach AGYW at heightened risk; however, in that study, fewer venue types were included and differences in observed risks across venue types were more substantial [22]. Previous work also suggested that respondent driven sampling may be used successfully to recruit vulnerable adolescents across Sub-Saharan Africa, and future comparisons of the differences between TLS and RDS methods to reach at-risk AGYW should be considered [14, 54].

In this study, AGYW vulnerability and HIV risk were high across venues sampled. Migration was a common source of heightened vulnerability and engagement in transactional sex was reported by AGYW across venue types. AGYW engaged in sex work may be the most vulnerable [55], but exclusive targeting of HIV risk reduction programs and interventions at traditional sex work hotspots may miss a number of other venue types where AGYW vulnerability is high, including AGYW engaged in transactional sex and other high risk sex. Data from a previous Ethiopian AGYW study among migrants supports the idea of the need for early intervention across venue types, as results suggested that AGYW often entered the workforce at locations in which engagement in condomless sex was less common, but due to poor working conditions, sexual abuse, or exploitation often transitioned into sex work or high risk sexual behaviors, including transactional sex, multiple sexual partners, or limited condom use, soon after [8].

Together these results suggest that local identification of potential venues where AGYW frequently congregate, alongside rapid assessments at the venues to verify presence of out-of-school AGYW, may identify venues which may not traditionally be thought of as high priority, but are locations in which many AGYW with substantial vulnerabilities may be reached. HIV prevention programs may utilize this general approach to engage AGYW along a continuum of heightened risk in mobile delivery of HIV prevention and testing services, STI screening and treatment, and family planning services, or to link AGYW at these sites to their services.

The interpretation of these study results should be completed in the context of several limitations. Firstly, as TLS samples from venues, the probability of inclusion is dependent upon the frequency of visiting the venues sampled. Safety and legality restricted accessibility to certain venue types such as shisha and khat bars (i.e. places where customers communally smoke or chew forms of tobacco). TLS does not identify private residences and, thus, AGYW working domestically were not recruited and married women may be under-represented. Additionally, due to the large number of sites—nearly 2500, unique AGYW venues identified in Addis Ababa—it was not feasible within this study to verify all venues. However, the validation of a subset of venues was in alignment with PLACE approaches [13]. The sampling strategy ensured proportional representation of identified venue types and accounted for how many times a KI mentioned a venue to ensure inclusion of key venues. Even with proportional representation of identified venue types, however, the proportion enrolled to screened varied by venue type, potentially leading to over- or underrepresentation of AGYW from certain venue types. For example, almost a third of participants were recruited from factory and construction sites, further reinforcing the importance of determining a maximum number of possible recruits from any one venue during data collection. Finally, the study was designed to assess vulnerability within Addis Ababa and thus sub-group analyses across venue types were in some cases underpowered. Nevertheless, the general trends across venues and the ability of utilizing a venue-based approach to reach a diverse sample of out-of-school vulnerable AGYW was supported.

## Conclusion

Reaching vulnerable AGYW from multiple entry points is essential to alter the course of the epidemic among young women. Venue-based TLS offers a promising method for identifying and recruiting vulnerable, out-of-school AGYW in Ethiopia. Vulnerable AGYW at high risk for HIV were reached, and significant vulnerabilities among AGYW across venue types observed, including venues and AGYW not commonly considered in hotspot recruitment approaches. Future research and programming efforts may benefit from considering community-informed venue-based methods which reach a diverse sample of vulnerable AGYW.

## Electronic supplementary material

Below is the link to the electronic supplementary material.

Supplementary material 1 (DOCX 15 kb)
