# Supplementary material for: Leveraging Geospatial Approaches to Characterize the HIV Prevention and Treatment Needs of Out-of-School Adolescent Girls and Young Women in Ethiopia
Source: AIDS Behav. 2019 May 27;23(Suppl 2):183–93. doi: 10.1007/s10461-019-02537-1 (PMC6773675; doi:10.1007/s10461-019-02537-1)
Supplement: Supplementary file 1 — Supplementary material 1 (DOCX 15 kb) [file 10461_2019_2537_MOESM1_ESM.docx]

**Supplemental Table I. Enrollment of adolescent girls and young women (AGYW) by venue type, Addis Ababa, 2018**

| **Venue type** | **No. of eligible venues visited** | **Total no. of AGYW enrolled** | **Average no. of AGYW attending site during recruitment** | **Average no. of AGYW Screened** | **Average no. of eligible AGYW** | **Average no. of enrolled AGYW** | **Proportion of enrolled to screened** |
| --- | --- | --- | --- | --- | --- | --- | --- |
| Bar, restaurant | 9 | 75 | 14 | 13 | 10 | 9 | 62.0% |
| Hotel, hostel, guest house | 12 | 112 | 15 | 15 | 12 | 10 | 61.2% |
| Brokers place | 8 | 81 | 27 | 21 | 17 | 10 | 48.2% |
| Street, street corner, street market, transportation centers | 16 | 148 | 53 | 25 | 18 | 9 | 37.1% |
| Construction site, shed, factory | 23 | 254 | 86 | 26 | 20 | 11 | 42.2% |
| Special villages | 3 | 33 | 50 | 31 | 22 | 11 | 35.1% |
| Youth center and training center | 7 | 67 | 30 | 19 | 16 | 10 | 49.3% |
| Other | 3 | 30 | 111 | 61 | 44 | 10 | 16.4% |
| **Total** | **81** | **800** | **50** | **23** | **18** | **10** | **42.4%** |
|  |  |  |  |  |  |  |  |
